# Adjuvant versus early salvage radiotherapy: outcome of patients with prostate cancer treated with postoperative radiotherapy after radical prostatectomy

**DOI:** 10.1186/s13014-019-1391-0

**Published:** 2019-11-11

**Authors:** Marco M. E. Vogel, Kerstin A. Kessel, Kilian Schiller, Michal Devecka, Jürgen E. Gschwend, Wilko Weichert, Jan J. Wilkens, Stephanie E. Combs

**Affiliations:** 1 Department of Radiation Oncology, Klinikum rechts der Isar, Technical University of Munich (TUM), Munich, Germany; 2 Institute for Radiation Medicine (IRM), Department of Radiation Sciences (DRS), Helmholtz Zentrum München, Neuherberg, Germany; 3 Deutsches Konsortium für Translationale Krebsforschung (DKTK), Partner Site Munich, Munich, Germany; 4 Department of Urology, Klinikum rechts der Isar, Technical University of Munich (TUM), Munich, Germany; 5 Institute of Pathology, Technical University of Munich (TUM), Munich, Germany

**Keywords:** Prostatic carcinoma, Postoperative radiation therapy, Biochemical relapse, Biochemical relapse free survival time, ART, SRT

## Abstract

**Background:**

Adjuvant (ART) and salvage radiotherapy (SRT) are two common concepts to enhance biochemical relapse free survival (BCRFS) in patients with prostate cancer (PC). We analyzed differences in outcome between ART and SRT in patients with steep decline of PSA-levels after surgery to compare outcome.

**Methods:**

We evaluated 253 patients treated with postoperative RT with a median age of 66 years (range 42–85 years) treated between 2004 and 2014. Patients with additive radiotherapy due to PSA persistence and patients in the SRT group, who did not achieve a postoperative PSA level <0.1 ng/mL were excluded. Hence, data of 179 patients was evaluated. We used propensity score matching to build homogenous groups. A Cox regression model was used to determine differences between treatment options. Median follow-up was 32.5 months (range 1.4–128.0 months).

**Results:**

Early SRT at PSA levels <0.3 ng/mL was associated with significant longer BCRFS than late SRT (HR: 0.32, 95%-CI: 0.14–0.75, *p* = 0.009). Multiple Cox regression showed pre-RT PSA level, tumor stage, and Gleason score as predictive factors for biochemical relapse. In the overall group, patients treated with either ART or early SRT showed no significant difference in BCRFS (HR: 0.17, 95%-CI: 0.02–1.44, *p* = 0.1). In patients with locally advanced PC (pT3/4) BCRFS was similar in both groups as well (HR: 0.21, 95%-CI:0.02–1.79, *p* = 0.15).

**Conclusion:**

For patients with PSA-triggered follow-up, close observation is essential and early initiation of local treatment at low PSA levels (<0.3 ng/mL) is beneficial. Our data suggest, that SRT administered at early PSA rise might be equieffective to postoperative ART in patients with locally advanced PC. However, the individual treatment decision must be based on any adverse risk factors and the patients’ postoperative clinical condition.

**Study registration:**

The present work is approved by the Ethics Commission of the Technical University of Munich (TUM) and is registered with the project number 320/14.

## Background

Although the recent ProtecT trial [1] showed no difference in outcome for patients treated either with surgery or radiotherapy (RT), radical prostatectomy (RP) is still the treatment option mostly chosen by patients with prostate cancer (PC) [2]. However, studies showed that approximately one-third to one-half [3] of the patients develop a biochemical relapse (BCR), which calls for treatment options e.g. postoperative local RT. Two postoperative approaches to reduce risk for relapse are commonly used: Adjuvant radiotherapy (ART), which should be performed within 4 months after surgery, triggered mainly by tumor size and surgical margins, and salvage radiotherapy (SRT), which is performed when prostate-specific antigen (PSA) levels increase during follow-up [4]. The term additive radiotherapy is used when RT is applied on basis of a persistence of PSA levels (most commonly PSA >0.1 ng/mL) after surgery.

Three large trials (EORTC 22911 [5, 6], SWOG 8794 [7, 8] and ARO [9–11]) with over 1700 patients in total showed a benefit for ART in biochemical relapse free survival (BCRFS) compared to observation. In all three trials, ART was compared to RP alone with a following wait-and-see policy.

Up to this point, SRT has only been examined in retrospective cohort studies or meta-analyses. Song et al. [12] and Stephenson et al. [13] investigated the oncological outcome of SRT. Song et al. showed a 5-year-BCRFS of 53.6%, while Stephenson et al. published a 6-year-BCRFS of 32.0%. Trock et al. [14] compared SRT with and without androgen-deprivation therapy (ADT) to patients treated with observation only. SRT was associated with a 3-times higher PC specific survival.

Since there is an obvious lack of data comparing ART to SRT directly, there is an ongoing debate on whether SRT is equal to ART. Budiharto et al. [15] evaluated patients with high-risk PC and showed a benefit for ART in this patient group. Briganti et al. [16] analyzed patients with pT3N0 R0-R1 tumors and found no differences in outcome.

Results of three randomized prospective trials on this topic are still on the way: the RAVES study [17] (ClincialTrials.gov Identifier: NCT00860652), the RADICALS trial (ClincialTrials.gov Identifier: NCT00541047) and the GETUG-17 trial (ClincialTrials.gov Identifier: NCT00667069). First results are expected in 2021. We previously reported data on toxicity in a patient cohort comparing immediate postoperative RT versus SRT [18]. In the present article we evaluate the oncological outcome after ART compared to SRT in the same cohort to answer the question whether SRT is equieffective to ART in terms of oncological outcome.

## Methods

We retrospectively evaluated 253 patients with a median age of 66 years (range 42–85 years). Patients were treated at the Department of Radiation Oncology, Klinikum rechts der Isar, Technical University of Munich (TUM), Munich, Germany, between 2004 and 2014. ART was defined as RT within 6 months after surgery or in exceptional cases longer due to delayed start of RT because of postoperative side effects (e.g. urine incontinence). One patient in the ART group did not complete RT (total dose 52.0 Gy) due to severe pain caused by an anal fissure, which occurred pre-RT. SRT was defined as postoperative RT after 6 months and BCR with post-RT PSA level <0.1 ng/mL. Additive RT was defined as RT due to PSA persistence with PSA level ≥ 0.1 ng/mL after surgery.

**Fig. 1:**
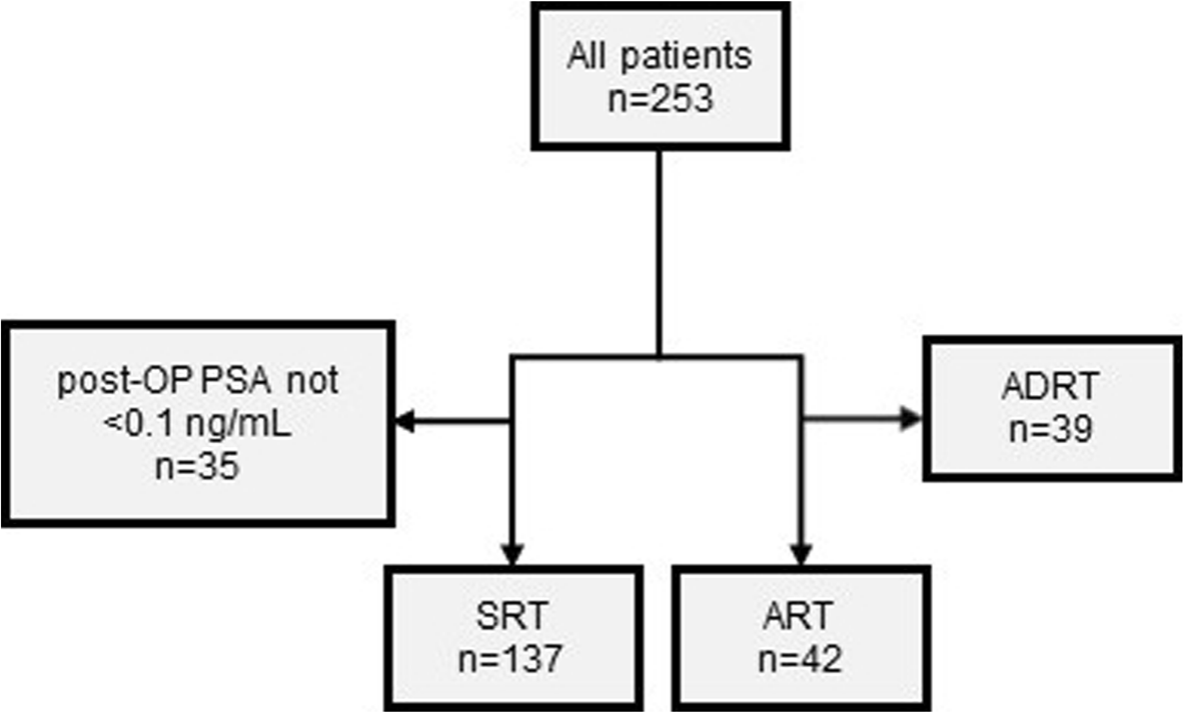
Flow chart of included patients. Patients with SRT who did not achieve a postoperative PSA level <0.1 ng/mL or received additive radiotherapy were excluded from analysis. (SRT Salvage radiotherapy, ART Adjuvant radiotherapy, ADRT Additive radiotherapy, PSA Prostate-specific antigen, OP Surgery)

Of all patients, 42 received ART (median time after RP: 4.4 months, range: 2.2–9.9 months), while SRT (median time after RP: 35.7 months, range: 5.7–200.1 months) was administered in 137 patients. Additive RT due to PSA persistence was given to 39 patients and were excluded from ART group. Thirty-five patients formally received salvage treatment but did not achieve a postoperative PSA level <0.1 ng/mL. Those patients were excluded, due to persistent PSA levels. The flow chart is shown in Fig. 1.

The primary endpoint was BCR after RT. BCR was defined as a post-RT PSA level >0.2 ng/mL after reaching the post-RT PSA nadir. Missing data and further follow-up were acquired by contacting patients via letter and/or phone. Before study initiation, ethical approval was obtained from the ethics committee of the Technical University of Munich (TUM), Germany (Medical Faculty, project number: 320/14).

ROC (Receiver Operating Characteristic) analysis was used to determine cut-off values for early salvage radiotherapy. We used adjusted Cox regression to compare BCRFS in both groups. Only patients without ADT (*n* = 111/137) were included in this sub-analysis. For further evaluation ART (*n* = 21) was solely compared to early SRT (*n* = 64) without ADT. We used propensity score matching (PSM) to build homogenous groups. Cox regression analysis was used to determine BCRFS. All other statistical analyses were performed descriptively with exploratory intention using proportions, means (range), and 95%-confidence intervals (95%-CI). A *p*-value <0.05 was considered as statistically significant. For all evaluations, we used SPSS version 21 (IBM, Armonk, USA).

## Results

Based on the above-mentioned criteria, we included 179 patients in this evaluation. Patient characteristics are shown in Table 1.

**Table 1:** Patients characteristics

		All (*n* = 179)	ART (*n* = 42)	SRT (*n* = 137)
Age [years]		67 (range: 42–85)	67 (range: 42–75)	66 (range: 49–85)
Initial PSA level [ng/mL] [19]				
Low risk (< 10)		111 (62.0%)	22 (52.4%)	89 (65.0%)
Intermediate risk (10–20)		35 (19.6%)	10 (23.8%)	25 (18.2%)
High risk (> 20)		30 (16.8%)	10 (23.8%)	20 (14.7%)
Missing		3 (1.7%)	0 (0.0%)	3 (2.2%)
ISUP Grading (Gleason score) [20]				
Group 1 (≤ 6)		26 (14.5%)	2 (4.8%)	24 (17.5%)
Group 2 (3 + 4 = 7)		53 (29.6%)	8 (19.0%)	45 (32.8%)
Group 3 (4 + 3 = 7)		45 (25.1%)	14 (33.3%)	31 (22.6%)
Group 4 (8)		27 (15.1%)	6 (14.3%)	21 (15.3%)
Group 5 (≥ 9)		24 (13.4%)	12 (28.6%)	12 (8.8%)
Missing		4 (2.3%)	0 (0.0%)	4 (2.9%)
Postoperative T-stage [19]				
Low risk	pT2a	16 (8.9%)	0 (0.0%)	16 (11.7%)
Intermediate risk	pT2b	9 (5.0%)	1 (2.4%)	8 (5.8%)
	pT2c	56 (31.3%)	4 (9.5%)	52 (38.0%)
High risk	pT3a	46 (25.7%)	17 (40.5%)	29 (21.2%)
	pT3b	46 (25.7%)	20 (47.6%)	26 (19.0%)
	pT4	6 (3.4%)	0 (0.0%)	6 (4.4%)
Postoperative nodal status				
Negative (pN0)		157 (87.7%)	29 (69.0%)	128 (93.4%)
Positive (pN1)		22 (12.3%)	13 (31.0%)	9 (6.6%)
Surgical margins				
R0		93 (52.0%)	10 (23.8%)	83 (60.6%)
R1		86 (48.0%)	32 (76.2%)	54 (39.4%)
Omission of ADT (additive or adjuvant ADT)		35 (19.6%)	9 (21.4%)	26 (19.0%)
Median time of ADT [months]		13 (range:1–140)	17 (range: 4–32)	13 (range: 1–140)
RT technique				
3D-CRT		37 (20.7%)	8 (19.0%)	29 (21.1%)
Dynamic IMRT		10 (5.6%)	1 (2.4%)	9 (6.6%)
VMAT		113 (63.1%)	30 (71.4%)	83 (60.6%)
Helical IMRT		19 (10.6%)	3 (7.2%)	16 (11.7%)
Median total dose [Gy]		64.0 (range: 52.0–70.2)	60.0 (range: 52.0–64.8)	64.0 (range: 59.4–70.2)
Median follow-up [months]		32.5 (range: 1.4–128.0)	36.5 (range: 1.4–102.5)	31.5 (range: 1.5–128.0)

Risk classification according to National Comprehensive Cancer Network guidelines [19]. Gleason score grading in groups according to the 2014 International Society of Urological Pathology (ISUP) Consensus Conference [20]. (*ART* Adjuvant radiotherapy, *SRT* Salvage radiotherapy, *PSA* Prostate-specific antigen, *ADT* Androgen deprivation therapy, *3D-CRT* Three-dimensional conventional radiotherapy, *IMRT* Intensity modulated radiotherapy, *VMAT* Volumetric intensity modulated arc therapy)

Median pre-RT PSA level for ART was below detection limit with 0.04 ng/mL (range: 0.00–0.08 ng/mL) and for SRT 0.29 ng/mL (range: 0.02–10.0 ng/mL). A median total dose of 64.0 Gy (range: 52.0–70.2 Gy) was delivered with single doses of 1.8–2.14 Gy. Overall median follow-up was 32.5 months (range 1.4–128.0 months). In ART and SRT group 10 and 22 patients received additional irradiation to the pelvic lymph nodes. Table 2 shows rates of biochemical relapse and occurrence of metastases for patients with ART and SRT in overall group.

**Table 2:** Rates of biochemical relapse and occurrence of metastases for patients with ART and SRT in overall group

	Biochemical relapse		Occurrence of metastases	
	ART (*n* = 5)	SRT (*n* = 76)	ART (*n* = 5)	SRT (*n* = 76)
Locally confined PC (pT2)	0 (0.0%)	18 (23.7%)	0 (0.0%)	9 (11.8%)
	ART (*n* = 37)	SRT (*n* = 61)	ART (*n* = 37)	SRT (*n* = 61)
Locally advanced PC (pT3/4)	4 (10.8%)	26 (42.6%)	1 (2.7%)	12 (19.7%)
	ART (*n* = 42)	SRT (*n* = 137)	ART (*n* = 42)	SRT (*n* = 137)
Overall group	4 (9.5%)	44 (31.1%)	1 (2.4%)	21 (15.3%)

*ART* Adjuvant Radiotherapy, *SRT* Salvage radiotherapy, *PC* Prostate cancer

### Early versus late salvage radiotherapy (SRT)

**Fig. 2:**
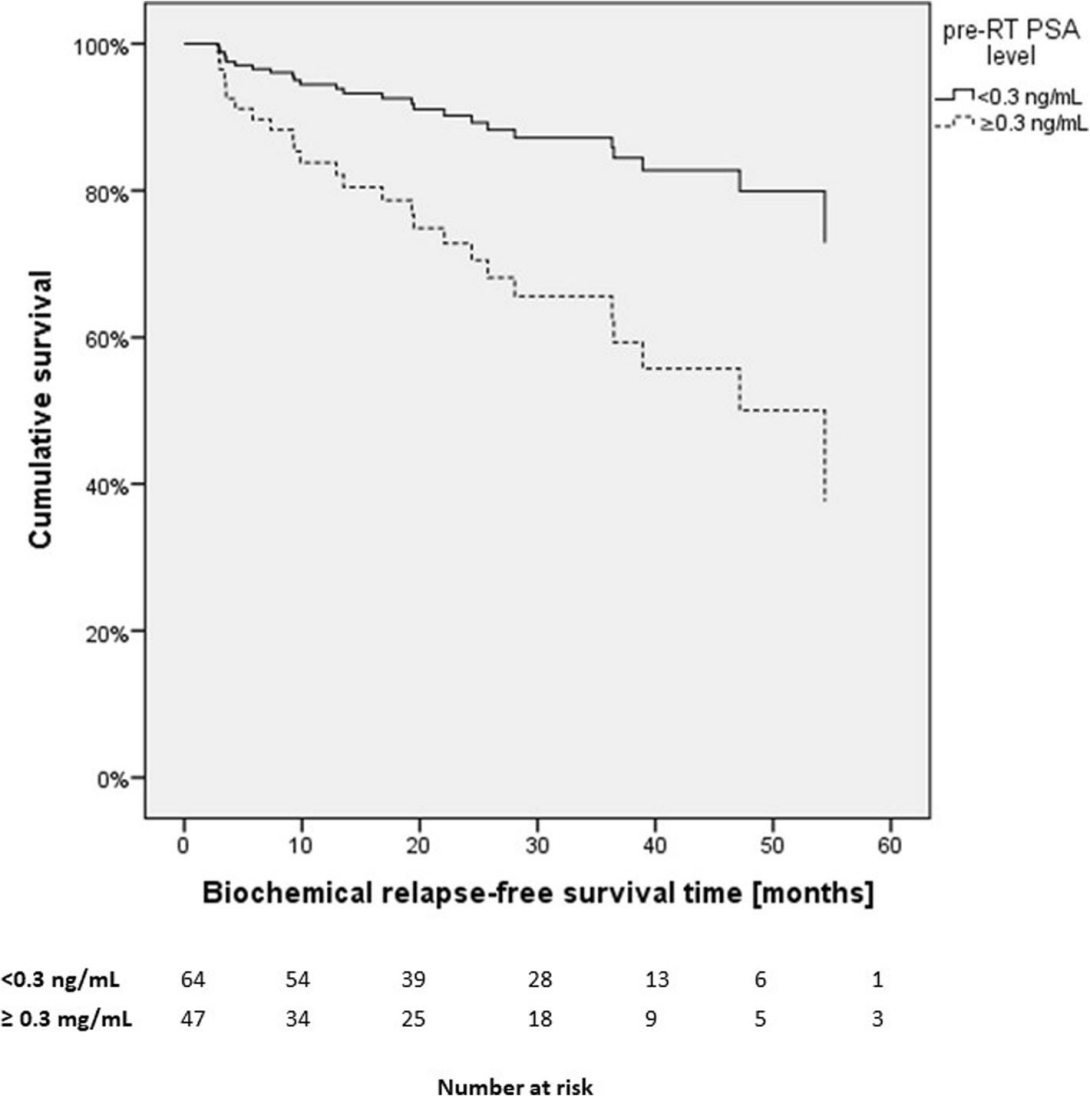
Cox regression for early (PSA <0.3 ng/mL) versus late SRT (PSA ≥0.3 ng/mL) for patients without androgen deprivation therapy adjusted for tumor stage, nodal status, surgical margins and Gleason score (RT Radiotherapy, PSA Prostate-specific antigen)

Data of 111 patients was used. ROC analysis determined a PSA of 0.3 ng/mL as a cut-off value, which resulted in 64 patients in early and 47 patients in late SRT group. We compared BCRFS of early SRT (PSA <0.3 ng/mL) and late SRT (PSA ≥0.3 ng/mL) with Cox regression adjusted for tumor stage (≤T2c vs. ≥T3a), nodal status (N0 vs. N1), Gleason score (≤7a vs ≥7b), and surgical margins (R0 vs. R1). BCRFS in both groups (<0.3 ng/mL versus ≥0.3 ng/mL) was significantly different (HR: 0.32, 95%-CI: 0.14–0.75, *p* = 0.009) (Fig. 2). Univariate Cox regression showed significance for pre-RT PSA level, tumor stage, and Gleason score. In multiple analysis all three variables remained independent predictive factors for early biochemical relapse (Table 3).

**Table 3:** Cox regression for early versus late SRT

Factors	Univariate Cox regression			Multiple Cox regression		
	HR	95%-CI	*p*	HR	95%-CI	*p*
Group (<0.3 ng/mL versus ≥0.3 ng/mL)	0.34	0.15–0.76	0.009*	0.30	0.13–0.69	0.004*
Tumor stage (≤T2c vs. ≥T3a)	0.37	0.17–0.80	0.01*	0.43	0.19–0.97	0.04*
Nodal status (N0 vs. N1)	0.40	0.09–1.72	0.22			
Gleason score (≤7a vs ≥ 7b)	0.34	0.15–0.78	0.01*	0.35	0.15–0.84	0.02*
Surgical margins (R0 vs. R1)	1.37	0.61–3.05	0.44			

Univariate and multiple Cox regression for predictive factors for biochemical relapse-free survival for early (PSA <0.3 ng/mL) versus late SRT (PSA ≥0.3 ng/mL) (*ART* Adjuvant radiotherapy, *SRT* Salvage radiotherapy, *HR* Hazard ratio, *95%-CI* 95%-confidence interval, * = significant result)

### Overall group

For analysis of outcome, we only evaluated patients without ADT which resulted in 21 and 64 cases in ART and early SRT group, respectively. Before PSM, tumor stage and surgical margins showed significant differences in both groups. Therefore, we applied PSM for the two variables. Tumor characteristics before and after PSM are shown in Table 4. Sample size of patients with locally confined tumors was too small to report outcome analysis.

**Table 4:** Tumor characteristics before and after propensity score matching

	Before PSM			After PSM		
	ART n = 21	Early SRT n = 64	*p*	ART n = 21	Early SRT n = 21	*p*
Tumor stage						
≤T2c	4 (19.0%)	39 (60.9%)	0.001*	4 (19.0%)	0 (0.0%)	0.11
≥T3a	17 (81.0%)	25 (39.1%)		17 (81.0%)	21 (100.0%)	
Nodal status						
N0	20 (95.2%)	63 (98.4%)	0.44	20 (95.2%)	20 (95.2%)	1.00
N1	1 (4.8%)	1 (1.6.%)		1 (4.8%)	1 (4.8%)	
Gleason score						
≤7a	6 (28.6%)	31 (48.4%)	0.13	6 (28.6%)	9 (42.9%)	0.52
≥7b	15 (71.4)	31 (48.4%)		15 (71.4)	12 (57.1%)	
Missing	0 (0.0%)	2 (3.1%)		0 (0.0%)	0 (0.0%)	
Surgical margins						
R0	5 (23.8%)	39 (60.9%)	0.005*	5 (23.8%)	12 (57.1%)	0.06
R1	16 (78.2%)	25 (39.1%)		16 (78.2%)	9 (42.9%)	
Biochemical relapse						
no	20 (95.2%)	55 (85.9%)	n./a.	20 (95.2%)	16 (76.2%)	n./a.
yes	1 (4.8%)	9 (14.1%)		1 (4.8%)	5 (23.8%)	

*PSM* Propensity score matching, *ART* Adjuvant Radiotherapy, *SRT* Salvage radiotherapy, *T* Tumor stage, *N* Nodal status, *R* Surgical margin, * = significant result, *n./a.* Not applicable

**Fig. 3:**
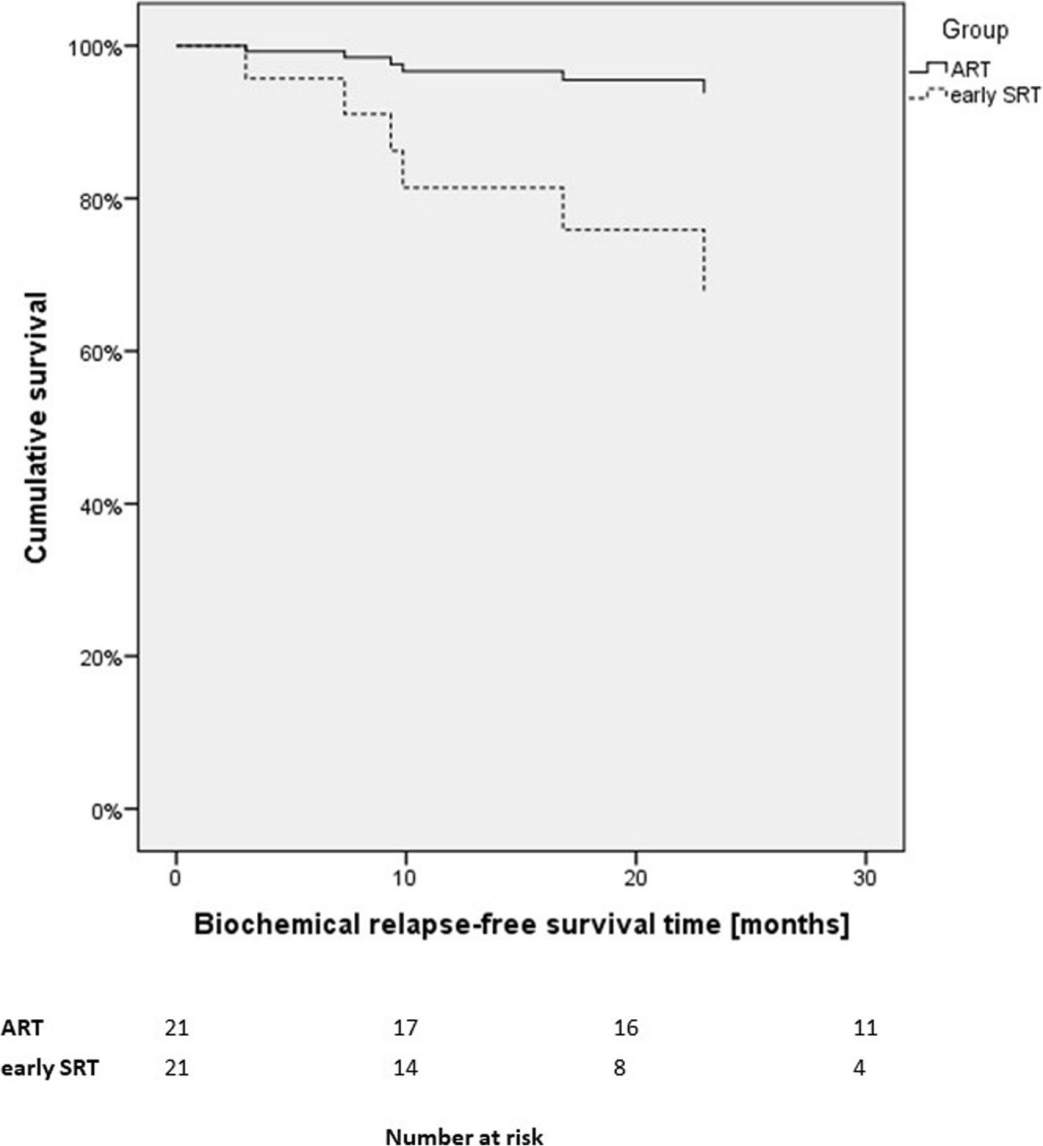
Cox regression of biochemical relapse-free survival for patients in overall group (ART Adjuvant radiotherapy, SRT Salvage radiotherapy)

We built 21 pairs with patients of ART and early SRT group. BCRFS (see Fig. 3) was not significantly different between both groups (HR: 0.17, 95%-CI: 0.02–1.44, *p* = 0.1).

### Locally advanced prostate Cancer (pT3/4)

**Fig. 4:**
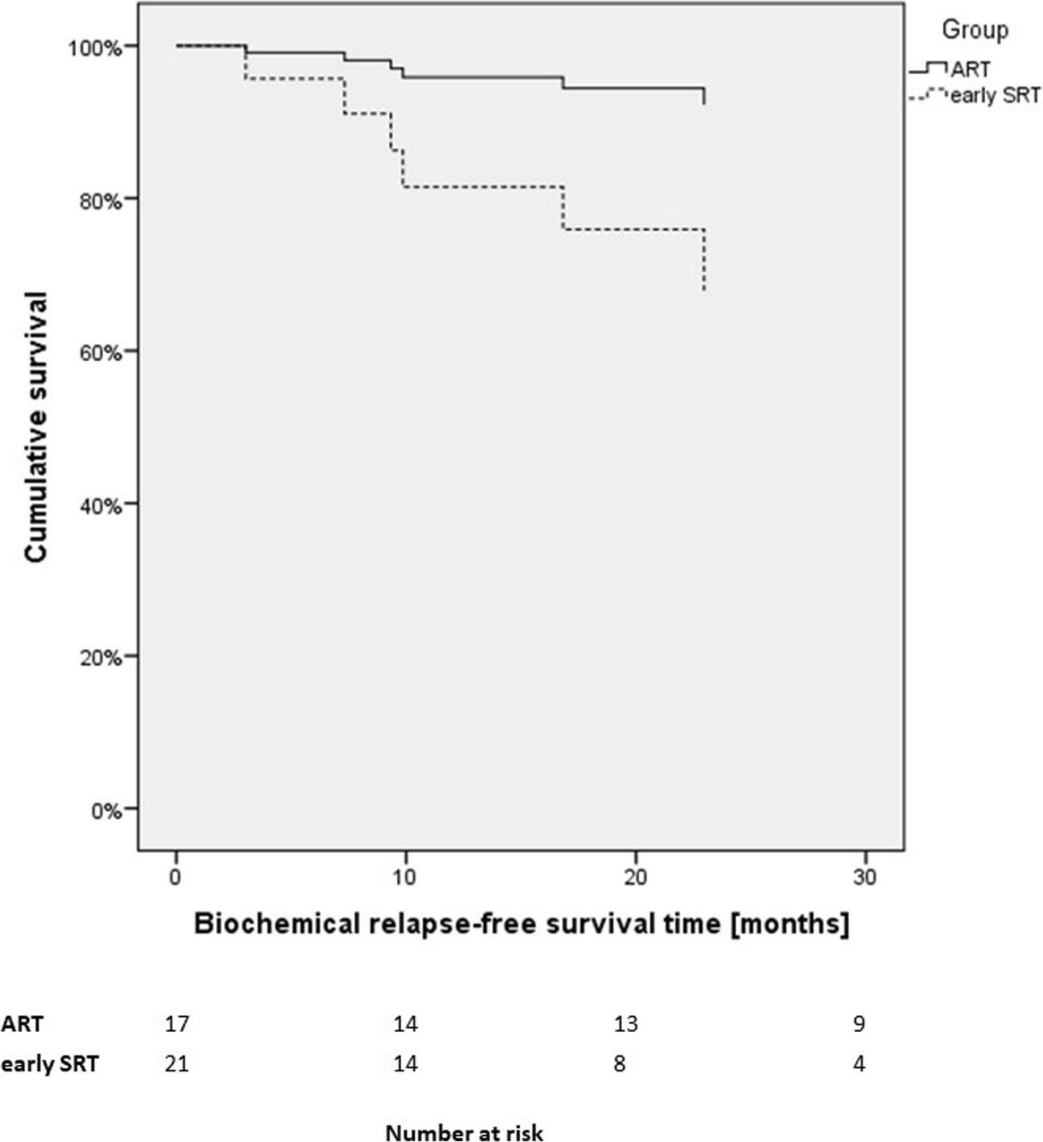
Cox regression of biochemical recurrence-free survival for patients with pT3/4 tumors (ART Adjuvant radiotherapy, SRT Salvage radiotherapy)

For patients with locally advanced PC Cox regression showed no significant difference in BCRFS (see Fig. 4) of ART versus early SRT (HR: 0.21, 95%-CI:0.02–1.79, *p* = 0.15).

## Discussion

Postoperative RT is a common approach with the goal to prolong BCRFS in patients who previously underwent RP. The question whether SRT versus ART is equieffective is still controversial. Our results suggest, that when early SRT at PSA levels <0.3 ng/mL is administered, patients show a significantly better BCRFS with a 68% reduced risk for BCR. Pre-RT PSA level, tumor stage, and Gleason score remained significant predictors in multiple Cox regression. However, no significant difference for patients receiving ART or early SRT in the overall group was seen. We couldn’t determine a difference in BCRFS in the subgroup analysis of patients with locally advanced (pT3/4) as well. For evaluation of locally confined tumors the sample size was too small.

In the past, three trials (EORTC 22911 [5, 6], ARO 96–02 [9–11], and SWOG 8794 [7, 8]) showed a significant benefit for ART compared to a wait-and-see strategy. Bolla et al. showed a BCRFS at 10 years of 60.6 and 41.1%, respectively. Wiegel et al. stated a BCRFS at 5 years of 77% for ART and 54% for a wait-and-see strategy. At 10 years, progression-free survival was 56% versus 35%, respectively. Thompson et al. showed a median BCRFS of 10.3 years for ART and 3.1 years for the wait-and-see group. Here, the primary endpoint was MFS, which accumulated to a median of 14.7 years for ART and 13.2 years for the wait-and-see group. However, Arcangeli et al. [21] performed a critical review of the three randomized trials and showed that in two of the three trials (SWOG 8794 and EORTC 22911) a proportion of patients had a detectable PSA and therefore received formally SRT rather than ART. Further, used doses are considered as inadequate, nowadays. Up to half of the patients in the observational arm received SRT at PSA above 1 ng/mL, which is considered inappropriate, nowadays. Moreover, only the SWOG 8794 trial showed an effect on overall survival.

SRT was only evaluated retrospectively, so far. Song et al. [12] determined a 6-year BCRFS of 32.0% for patients receiving SRT. Significant predictive factors for BCR were pre-RT PSA level ≥1.0 ng/mL, tumor stage ≥T3a, Gleason score ≥7, PSA doubling time <12 months and no visible lesion on pelvic MRI. In line with the described study, a high Gleason score was a predictive factor in our evaluation. Besides Gleason score ≥7b, ≥T3a-tumors were also significantly associated with BCR in the present study. Therefore, especially patients with high risk tumor features should be treated without delay with SRT in case of rising PSA levels. Trock et al. [14] compared observation only to SRT with and without ADT. A benefit for SRT was shown, while ADT had no influence on BCRFS. This remains surprising: ADT as gonadotropin-releasing hormone agonists/antagonists, and antiandrogens reduce the release or function of testosterone and therefore prevent the tumor cells from growth and release of PSA [22]. Consequentially, ADT prolongs BCRFS to the point of castration resistance. Recently, Shipley et al. [23] evaluated SRT with ADT versus placebo. Results showed that patients with additional administration of 24 months of ADT had a significantly better overall survival and a significantly lower rate of distant metastases and death from PC. However, the data of Shipley et al. suggest, that especially patients with pre-RT PSA levels >0.7 ng/mL benefit from addition of ADT. In an earlier study, Carrie et al. compared SRT alone versus SRT with addition of 6 months of ADT and found a significant benefit for the addition of ADT [24]. In patients with ART, ADT must be considered when a positive nodal status is present [25, 26].

To our knowledge all comparative studies of ART versus SRT to date are of retrospective nature. Selected studies are shown in Table 5. Five of the presented series (Budiharto et al. [15], Jereczek-Fossa et al. [27], Ost et al. [28], Mishra et al. [29] and Detti et al. [30]) showed a significant benefit regarding BCRFS in the ART group. However, after Mishra et al. [29] incorporated propensity score calculation in their data, there was only a trend towards significance in BCRFS. Jereczeck-Fossa et al. [27] stated no statistically significant difference in MFS in their cohort. Briganti et al. [16] and Fossati et al. [31] showed an equal effect on the oncological outcome. In comparison to the other series, Briganti et al. [16] only included pT3N0 tumors with positive and negative surgical margins. Further, Fossati et al. and Briganti et al. investigated early SRT with start of RT at PSA levels ≤0.5 ng/mL while all other series were not purely focused on RT at low PSA levels. Our data suggest as well, that patient with locally advanced tumors show similar outcome, when treated with early SRT compared to ART.

**Table 5:** Comparison of selected previous series evaluating ART versus SRT

	n	BCRFS	*p*	Included tumors	BCR-criteria
Budiharto et al. [15]	ART: 130	5-years: 87% (95%-CI: 77–98%)	<0.001*	pT2-T4, N0, R0/R1	Not available
	SRT: 89	5-years: 34% (95%-CI: 11–56%)			
Jereczek-Fossa et al. [27]	ART: 258	4-years: 81.7%	<0.0001*	pT2-T4, N0, R0/1	(I) Post-RT PSA level nadir plus 0.1 ng/mL, or any PSA level greater than the pre-RT PSA level (II) Salvage-ADT
	SRT:173	4-years: 60.5%			
Briganti et al. [16]	ART: 390	2-years: 91.4% 5-years: 78.4%	0.9	pT3, N0, R0/1	Post-RT PSA level >0.2 ng/mL and rising
	SRT: 225	2-years: 92.8% 5-years: 81.8%			
Ost et al. [28]	ART: 144	3-years: 90%	0.002*	pT2-T4, N0, R0/1	Increase of more than 0.2 ng/mL above lowest post-OP PSA value
	SRT: 134	3-years: 65%			
Mishra et al. [29]	ART: 74	5-years: 84% 10-years: 73%	0.0001*	pT2, N0, R1 or pT3-T4, N0	For patients with undetectable pre-RT PSA: ≥0.4 ng/mL with a subsequent confirmation. For all other patients: (I) Three documented increases measured at least 6 weeks apart (II) Start of ADT
	SRT: 122	5-years: 55% 10-years: 41%			
Detti et al. [30]	ART: 203	BCR at time of analysis 20.7%,	0.03*	pT2-T4, N0, R0/1	Two consecutive measurements >0.2 ng/mL, measured at least 30 days apart.
	SRT: 104	BCR at time of analysis: 31.7%			
	n	MFS	*p*	Included tumors	Metastasis-criteria
Fossati et al. [31]	ART: 243	8-years: 92% (95%-CI: 87–93%)	0.9	pT3–4, N0, R0/1	Distant metastases in bones, parenchymal organs, or soft tissue.
	SRT: 267	8-years: 91% (95%-CI: 84–95%)			

*ART* Adjuvant radiotherapy, *SRT* Salvage radiotherapy, *RT* Radiotherapy, *BCRFS* Biochemical relapse-free survival time, *BCR* Biochemical relapse, *MFS* Metastasis-free survival time, *T* Tumor stage, *N* Nodal status, *R* Surgical margins, *ADT* Androgen deprivation therapy, *PSA* Prostate-specific antigen, *95%-CI* 95%-confidence interval, * = significant result

Since Stephenson et al. [13] showed a better outcome for patients receiving early SRT at PSA levels of 0.5 ng/mL or less the dictum of salvage treatment changed to “the earlier, the better” [32, 33]. The data of Bartkowiak et al. even advocates for a very early SRT at PSA levels of 0.2 ng/mL or less [34]. However, such low cut-off values are conflicting to the widely accepted definition of biochemical relapse after RP with two consecutive measurements of 0.2 ng/mL or higher [35]. Our data suggests an (very) early SRT at PSA levels less than 0.3 ng/mL. Therefore, close PSA monitoring remains an important follow-up strategy for patients after RP. The threshold of 0.3 ng/mL might be more beneficial in clinical routine, as well as in the discourse with patients. It must be kept in mind that our data derives from an era, where PSMA-PET imaging (Prostate-specific Membrane Antigen-Positron Emission Tomography) was not excessively used. In the last few years, PSMA-PET imaging has become an effective tool for staging and precise treatment of patients with BCR after RP [36, 37]. Whereas in the past, radiation oncologists had to administer empiric treatment to the prostate bed mostly without an imaging correlate, today the PSMA-PET accurately illustrates recurrent tumor sites in most cases. Nevertheless, negative PSMA-PET imaging shall not delay initiation of SRT [38], as discussed above early salvage treatment is crucial to good biochemical response. The perfect cut-off value of PSA indicating a high chance of visualization of tumor relapse in PSMA-PET imaging remains a topic of discussion. Perera et al. reported rates of 58% and 76% for PSA levels of 0.2–1.0 ng/mL and 1.0–2.0 ng/mL for PET scans with gallium-68 tracers [39]. However, the recently emerging use of fluorine-18 tracers might allow for better detection rates making the use of PSMA-PET imaging reasonable starting at PSA values as low as 0.2 ng/mL with a detection rate of 61.5% for patients with values between 0.2–0.5 ng/mL [40].

One point of criticism towards ART is the fact of possible overtreatment for patients who might never experience BCR. Previous series showed, that one-third to one-half [3] of the patients undergoing RP develop BCR. Patients receiving ART, that never might have relapsed, are exposed to possible toxicities and side effects caused by RT. In an earlier publication [18], we showed, that patients with immediate postoperative RT compared to SRT experience significantly higher rates of early gastrointestinal toxicities as proctitis, as well as early genitourinary side effects as urinary tract obstruction. Hence, the decision between ART or PSA-based follow-up and potential SRT should also be based on the patients’ postoperative clinical condition and any risk factors, as well as the patients’ preference. In terms of RT toxicity, patients may benefit from SRT with lower toxicity.

The European guidelines recommend discussion of ART in patients with pT3N0M0 tumors with high risk features such as positive surgical margins [4]. The German guideline recommends performing ART in patients with pT3N0M0 tumors with positive surgical margins (high grade of recommendation), pT3N0M0 tumors with negative surgical margins (moderate grade of recommendation) and pT2N0M0 tumors with positive surgical margins (low grade of recommendation) [38]. Positive surgical margins did not emerge as a predictive factor in our analysis. However, based on the previous results [15, 27–30] it remains discussable to use immediate postoperative RT depending on high risk tumor features such as tumor stage, positive surgical margins, high Gleason score, lymphovascluar invasion, perineural invasion, and high iPSA.

In comparison to all the mentioned series, we included patients with positive nodal status. ART in patients with intermediate to high risk tumors features and positive nodal status is reported to be beneficial [41]. However, no randomized data is published on this subject. Therefore, we suggest, that the decision on ART for patients with positive postoperative lymph node status should remain individual.

The median total doses of 60 Gy in the ART group and 64 Gy in the SRT group remain at the lower end of dosage given to the prostate bed, nowadays. In the last years, generally doses of 64–70 Gy are prescribed according to published data and guidelines [42]. The SAKK 09/10 trial currently compares dose-intensified SRT 64 Gy versus 70 Gy. The reported toxicity is low [43, 44], however data on outcome needs to be awaited.

The results of our study have limitations, as the data is of retrospective nature. We cannot account for the missing randomization: Patients receiving PSA-triggered SRT are negatively selected and might enter the study with a higher risk for BCR, while patients with no risk did not enter the analysis. Not all patients receiving RP are referred to the department of radiation oncology. Therefore, we cannot account for the referral practice. Moreover, patients who received ART might have never experienced a relapse. This being said, it is obvious that this flaw lies in the nature of the comparison and the only thing randomization would improve is the balance of the groups. The patient number and the limited follow-up time may be a further point of criticism. We cannot account for unknown covariates confounding the results. The tumor features (tumor stage, nodal status, surgical margins, Gleason score) differ in the ART and SRT group. Patients with high risk tumor features are more likely to be treated with ART as recommended in the guidelines. Therefore, we used PSM to deal with the imbalance. The heterogeneous definition for BCR after postoperative RT (see Table 5) remains a hurdle when comparing the data to other series. For primary RT, BCR is consistently defined by the Phoenix criteria [45]. The determination of BCR after postoperative RT remains difficult, hence, a consensual and consistent definition is desirable. Metastases were detected by imaging. However, no standardized follow-up imaging was performed with all patients as the data derives from the pre-PSMA-PET imaging era.

Up to date, three prospective trials are currently underway to determine whether ART and SRT are equieffective. The RAVES study [17] (ClincialTrials.gov Identifier: NCT00860652) is a randomized, multi-center phase 3 trial in Australia and New Zealand with 333 enrolled patients. The RADICALS trial (ClincialTrials.gov Identifier: NCT00541047) is a randomized, multicenter phase 3 study in the UK, Ireland, Denmark and Canada. Four thousand patients are expected to be included. Two studies are combined: In RADICALS RT patients with ART versus SRT are compared. In RADICALS HT, patients receiving RT with or without ADT are compared. The French GETUG-17 trial (ClincialTrials.gov Identifier: NCT00667069) is comparing ART versus SRT, both with concurrent ADT. Seven hundred eighteen patients shall be enrolled. The results of those prospective, randomized trials are eagerly awaited.

## Conclusion

The debate on postoperative RT for patients with PC remains controversial. Our data strongly advocates for initiation of SRT at low pre-RT PSA levels <0.3 ng/mL. Especially patients with tumor stage ≥T3a and Gleason score ≥7b should be treated rapidly. Our data suggests ART and early SRT at PSA levels <0.3 ng/mL to be equieffective, espcially in patients with locally advanced PC. However, we recommend to base the treatment decsision individually on the patients’ postoperative clinical condition and the tumor features, foremost tumor stage, nodal status, Gleason score and surgical margins.

## Data Availability

The datasets generated and analysed during the current study are not publicly available due regional data protection law but are available from the corresponding author on reasonable request.

## Abbreviations

(p)T: (Pathological) Tumor stage
3D-CRT: Three-dimensional conventional Radiotherapy
95%-CI: 95%-Confidence interval
ADRT: Additive radiotherapy
ADT: Androgen deprivation therapy
ARO: Arbeitsgruppe Radiologische Onkologie
ART: Adjuvant radiotherapy
BCR: Biochemical relapse
BCRFS: Biochemical relapse-free survival time
EORTC: European Organisation for Research and Treatment of Cancer
GETUG: Groupe d’étude des tumeurs urogénitales
Gy: Gray
HR: Hazard ratio
IMRT: Intensity modulated radiotherapy
ISUP: International Society of Urological Pathology
L: Lymphovascular invasion
M: Metastasis
N: Nodal status
ng/mL: Nanogram per milliliter
OP: Surgery
PC: Prostate cancer
PET: Positron emission tomography
PSA: Prostate-specific antigen
PSM: Propensity score matching
PSMA: Prostate-specific membrane antigen
R: Surgical margin
ROC: Receiver operating characteristic
RP: Radical prostatectomy
RT: Radiotherapy
SAKK: Schweizerische Arbeitsgemeinschaft für klinische Krebsforschung
SRT: Salvage radiotherapay
SWOG: Swedish Oncology Group
VMAT: Volumetric intensity modulated arc therapy
